# Whole-exome sequencing reveals novel variants of monogenic diabetes in Tunisia: impact on diagnosis and healthcare management

**DOI:** 10.3389/fgene.2023.1224284

**Published:** 2023-12-14

**Authors:** Nadia Kheriji, Hamza Dallali, Ismail Gouiza, Meriem Hechmi, Faten Mahjoub, Mehdi Mrad, Asma Krir, Manel Soltani, Hajer Trabelsi, Walid Hamdi, Afef Bahlous, Melika Ben Ahmed, Henda Jamoussi, Rym Kefi

**Affiliations:** ^1^ Laboratory of Biomedical Genomics and Oncogenetics, Institut Pasteur de Tunis, Tunis, Tunisia; ^2^ University of Tunis El Manar, Tunis, Tunisia; ^3^ Faculty of Medicine of Tunis, Tunis, Tunisia; ^4^ MitoLab Team, Unité MitoVasc, UMR CNRS 6015, Institut national de la santé et de la recherche médicale U1083, SFR ICAT, University of Angers, Angers, France; ^5^ Faculté de Médecine de Tunis, Research Unit UR18ES01 on “Obesity”, Tunis, Tunisia; ^6^ National Institute of Nutrition and Food Technology, Tunis, Tunisia; ^7^ Laboratory of Clinical Biochemistry and Hormonology, Institut Pasteur de Tunis, Tunis, Tunisia; ^8^ Laboratory of Clinical Immunology, Institut Pasteur de Tunis, Tunis, Tunisia

**Keywords:** whole-exome sequencing, bioinformatics analysis, 3D structural modeling, syndromic diabetes, genetic diagnosis, North African population, personalized medicine

## Abstract

**Introduction:** Monogenic diabetes (MD) accounts for 3%–6% of all cases of diabetes. This prevalence is underestimated due to its overlapping clinical features with type 1 and type 2 diabetes. Hence, genetic testing is the most appropriate tool for obtaining an accurate diagnosis. In Tunisia, few cohorts of MD have been investigated until now. The aim of this study is to search for pathogenic variants among 11 patients suspected of having MD in Tunisia using whole-exome sequencing (WES).

**Materials and methods:** WES was performed in 11 diabetic patients recruited from a collaborating medical center. The pathogenicity of genetic variation was assessed using combined filtering and bioinformatics prediction tools. The online ORVAL tool was used to predict the likelihood of combinations of pathogenic variations. Then, Sanger sequencing was carried out to confirm likely pathogenic predicted variants among patients and to check for familial segregation. Finally, for some variants, we performed structural modeling to study their impact on protein function.

**Results:** We identified novel variants related to MD in Tunisia. Pathogenic variants are located in several MODY and non-MODY genes. We highlighted the presence of syndromic forms of diabetes, including the Bardet–Biedl syndrome, Alström syndrome, and severe insulin resistance, as well as the presence of isolated diabetes with significantly reduced penetrance for Wolfram syndrome-related features. Idiopathic type 1 diabetes was also identified in one patient.

**Conclusion:** In this study, we emphasized the importance of genetic screening for MD in patients with a familial history of diabetes, mainly among admixed and under-represented populations living in low- and middle-income countries. An accurate diagnosis with molecular investigation of MD may improve the therapeutic choice for better management of patients and their families. Additional research and rigorous investigations are required to better understand the physiopathological mechanisms of MD and implement efficient therapies that take into account genomic context and other related factors.

## Introduction

Diabetes is one of the fastest growing global health emergencies of the 21st century. According to the 10th edition of the International Federation of Diabetes (IDF), 537 million adults aged between 20 and 79 years are currently living with diabetes (“IDF Diabetes Atlas 10th Edition” n.d.). In addition, this real burden is responsible for one death every 5 s. The Middle East and North Africa (MENA) region has the second highest rate in terms of diabetes prevalence, with a predicted increase of 87% between 2021 and 2045 (“IDF Diabetes Atlas 10th Edition” n.d.). The American Diabetes Association (ADA) classifies diabetes into four groups: type 1 diabetes (T1D), type 2 diabetes (T2D), gestational diabetes, and monogenic or atypical forms of diabetes (Care and Suppl, 2022). Monogenic diabetes (MD), as the name implies, results from a single gene rather than the contribution of multiple genes and environmental factors, as seen in T1D and T2D (Sanyoura, Philipson, and Naylor, 2018). It represents 3%–6% of all cases of diabetes. This prevalence is underestimated due to the misdiagnosis of MD as T1D or T2D (Shepherd et al., 2016). Thus, genetic investigation is essential to determine the MD class/subtype (Hattersley et al., 2018). There are currently more than 50 subtypes of MD, including neonatal diabetes mellitus (NNDM), maternally inherited diabetes and deafness (MIDD), maturity-onset diabetes of the young (MODY), rare diabetes-associated syndromic diseases, and other as yet unknown subclasses (Yeung et al., 2018). Each type is characterized by its phenotypic character, its causal gene, and its mode of transmission. Studies carried out in Europe, Asia, and the United States have reported a great deal of clinical, phenotypic, and genetic heterogeneity in MD (Mohan et al., 2018; Johnson and Jessica, 2019), while these forms of diabetes are still poorly known in the MENA region. In Tunisia, few cohorts of MD have been investigated until now. All these studies have shown that Tunisians have MD types different from those described in European populations. Indeed, among 89 patients screened for the most frequent mutations described in MD, only 13% were positive. This result suggests the involvement of other unidentified genes and variants describing different MD types with largely unknown genetic determinants among the Tunisian population.

The aim of this study is to identify genetic loci and causative mutations in Tunisian suspected MD patients using next-generation sequencing (NGS) technologies, namely, whole-exome sequencing “WES.” Through this study, we hope to aid clinicians in making better choices for the treatment of diabetic patients.

## Materials and methods

### Patients and sample collection

A total of 11 Tunisian suspected MD patients were recruited from the National Institute of Nutrition and Food Technology (INNTA) based on the following criteria:

➢ Hyperglycemia or diabetes mellitus recognized according to the latest American Diabetes Association guidelines as ≥1.26 g/L (7 mmol/L) (Care and Suppl, 2022)
➢ Young age of diabetes onset (≤40 years)
➢ Family history of diabetes in at least two generations
➢ Absence of or low pancreatic autoantibody titers

In order to take part in this study, patients, their parents, and participating family members gave their written informed consent. The study protocol was conducted according to the Declaration of Helsinki. It was approved by the Ethical Committee in Pasteur Institute of Tunis “IPT” (Registration numbers IRB00005445 and FWA00010074; ref. 2020/10/I/LR16IPT/V2).

Details about the medical history of the patients and their extended family members (where available) were documented using a questionnaire. In addition, clinical and metabolic data were collected from all patients, including the following details: demographic information, anthropometric measurements, diabetes history, and information related to treatment (oral anti-diabetic (OAD) and/or insulin injection).

Biochemical parameters, such as fasting plasma glucose (FPG), glycated hemoglobin (HbA1c), C-peptide, lipid profile [total cholesterol (TC), triglycerides (TG), high-density lipoprotein (HDL), and low-density lipoprotein (LDL)], C-reactive protein (CRP), and creatinine were measured in the Laboratory of Clinical Biochemistry and Hormonology in IPT.

Research for diabetes autoantibodies has been carried out in collaboration with the Laboratory of Clinical Immunology in IPT. In our study, glutamic acid decarboxylase (GAD), islet antigen 2 (IA2), and islet cell antibodies (ICAs) were analyzed in order to select suspected MD patients for genetic testing.

According to the literature, the antibody’s sensitivity as a screen criterion is still controversial. On the one hand, previous studies have shown that the presence of pancreatic autoantibodies makes the diagnosis of MD very unlikely, and genetic testing should not be performed (Mcdonald et al., 2011). On the other hand, recent studies have shown the coexistence of autoimmune diabetes and maturity-onset diabetes (MODY) in one patient (Donovan et al., 2022). In fact, 1%–2% of patients diagnosed with MODY had positive GAD antibodies (J. Urbanová et al., 2014). So, using negative antibodies as a screening method may not be practical without standardization. Thus, in this study, we chose to include examination of the autoantibody titers among MD subjects selected for genetic testing as reported by Jana Urbanová et al. (2013).

### Genomic investigation

• DNA extraction

We extracted genomic DNA from total blood using the FlexiGene DNA Kit (QIAGEN). Then, DNA quality was assessed using a NanoDrop spectrophotometer (Thermo Fisher Scientific) and DeNovix (Life Science Technologies).

• WES

We performed WES in collaboration with RAN Bi Links SARL (Carthagenomics) using the SureSelect Human All Exon V6 Kit (Agilent Technologies, CA, United States) and the Twist Human Core Exome Kit (Twist Biosciences). The captured libraries were sequenced on NovaSeq 6,000 System (Illumina, San Diego, CA, United States) to generate 151-bp paired-end reads.

• Bioinformatic analysis

The quality of the sequencing reads in FASTQ files was evaluated using FastQC (https://www.bioinformatics.babraham.ac.uk/projects/fastqc/), which was followed by adapter trimming using BBDuk (https://jgi.doe.gov/data-and-tools/bbtools/bb-tools-user-guide/bbduk-guide/). We aligned reads to the human reference genome hg38, and we subsequently called the genetic variants in a VCF file following the GATK best practices. Variant annotation was processed using ANNOVAR (Jiang et al., 2016). Prioritization of potential disease-causing variants was carried out in a set of 173 genes implicated in MD using the Variant Annotation and Filtering Tool (VarAFT) (Desvignes et al., 2018). The gene list was prepared through a literature review using PubMed (https:// www.ncbi.nlm.nih.gov/pubmed) and Mastermind (https://mastermind.genomenon.com/) [Supplementary Table S1].

In our study, all variants with minor allele frequency (MAF) > 0.01 in gnomAD (http://gnomad. broadinstitute.org) and GME (http://igm.ucsd.edu/gme/) databases were excluded. We kept non-synonymous, non-sense, frameshift, and splice site variants as they are more likely to have a functional impact. Non-synonymous variations were filtered to only retain those that were expected to be harmful by at least eight *in silico* pathogenicity prediction software tools. In addition, we selected variants predicted to alter splice sites by the Human Splicing Finder database (Desmet et al., 2009). Finally, we used Depth and Coverage Analysis (DeCovA) to evaluate the coverage of the genes that were selected (Dimassi et al., 2015). We screened different bioinformatics databases, such as PubMed (https://www.ncbi.nlm.nih.gov/pubmed), ClinVar (https://www.ncbi.nlm.nih.gov/clinvar/), VarSome (https://varsome.com/), and LOVD (https://www.lovd.nl), to identify prioritized variants and compare the clinical traits of the carriers and any genotype–phenotype correlations that had been found.

Additionally, we predicted the likelihood combinations of pathogenic variations using the machine-learning tool ORVAL (Oligogenic Resource for Variant AnaLysis: https://orval.ibsquare.be/). This website uses a variety of variants, genes, and gene pair biological features to make predictions and create networks of potential pathogenic variant combinations in gene pairs rather than isolated variations in individual genes. ORVAL offers the opportunity of an interactive exploration of the results to derive a biological explanation for patients. It provides a new crucial step in assisting researchers to better understand and investigate more complex genetic diseases (Renaux et al., 2019).

• Sanger sequencing

Sanger sequencing was performed to confirm the likely pathogenic predicted variants identified in patients to check for familial segregation. In short, using oligonucleotide primers designed by Primer3 software, genetic variations with exons were amplified from DNA samples using polymerase chain reaction (PCR). The ABI PRISM BigDye Terminator v3.1 Cycle Sequencing Kit was used to sequence the amplicons produced on the automated ABI3500 (Applied Biosystems, CA, United States) available in the technical platform in IPT. BioEdit software version 7.1 was used for the sequencing analysis (Hall, 1999).

•Structural modeling of the impact of newly identified variants

We studied the effect of some variants at the level of protein structure and function. The protein structures of INSR and GCKR were retrieved from the Protein Data Bank (PDB) (https://www.rcsb.org/) (PDB accession code: 7PG3 and 4BB9). For proteins that have not yet been explored by nuclear magnetic resonance spectroscopy (NMR spectrophotometry) or X-ray crystallography, we used the I-TASSER web server to generate a three-dimensional structure (3D). Since the I-TASSER web server is restricted to model protein chains with a maximum of 1,500 amino acids (aa), we created a partial 3D structure of proteins with a size less than 1,000 aa. Once energy minimization was performed on each predicted model using the YASARA minimization server (http://www.yasara.org/) (Krieger et al., 2009), MolProbity (http://molprobity.biochem.duke.edu/) and ProSA-web (https://prosa.services.came.sbg.ac.at/prosa.php) servers were applied to analyze the Ramachandran plot and to assess the quality of the obtained models, respectively. Then, the effect of variants on protein stability was assessed using mCSM (https://biosig.lab.uq.edu.au/mcsm/stability) and DUET (https://biosig.lab.uq.edu.au/duet/stability) servers. The Dynamut2 server (http://biosig.unimelb.edu.au/dynamut2/) was used to predict and visualize the interactions among aa residues, and the HOPE server (https://www3.cmbi.umcn.nl/hope/) was utilized to evaluate the size, hydrophobicity, and intramolecular interactions of both the wild-type and the mutant protein.

## Results

### Cohort study description

This study reports biochemical, immunological, and genetic results of 11 Tunisian suspected MD patients. The anthropometric and clinical characteristics of participants are summarized in Table 1. Biochemical measurements and immunological analysis results are reported in Table 2.

**TABLE 1:** Anthropometric and clinical characteristics of the 11 suspected MD patients.

Patient ID	Gender	Circumstance of diabetes discovery	Age at diagnosis of diabetes	Age at survey	Treatment	Family members with diabetes	Other clinical features
P1	M	SPUPD FPG = 19.08 mmol/L	27	31	OAD treatment for 4 years and then switch to insulin	5	Overweight and high TG
P2	F	SPUPD FPG = 22 mmol/L	14	17	OAD treatment and then switch to insulin + sulfonylurea (Irys)	3	Overweight, high TG, low HDL, abdominal pain, and vomiting
P3	F	SPUPD FPG = 27.8 mmol/L	7	8	Insulin	0	High acetone levels and normal weight
P4	M	SPUPD asthenia FPG = 33.4 mmol/L	5	6	Insulin	0	Absence of other clinical signs
P5	M	SPUPD asthenia FPG = 13.2 mmol/L	11	15	Insulin	1	Frequent hypoglycemia due to high insulin dose
P6	F	SPUPD FPG = 12.8 mmol/L	13	14	Insulin	0	Dumbness and deafness, mental retardation, delay in weight status, facial dysmorphia, and brachydactyly
P7	M	SPUPD HbA1C = 10%	8	13	Insulin	0	No ketoacidosis, nervous character, learning difficulties, and overweight
P8	M	SPUPD FPG = 16.7 mmol/L	21	24	Insulin + OAD and then remain on insulin only	0	Hearing loss, retinitis pigmentosa, and irritability
P9	F	SPUPD weight loss FPG = 22.2 mmol/L	24	25	Insulin	1	Frequent hypoglycemic and neurological problem
P10	M	FPG = 10.52 mmol/L HBA1c = 7.7%	15	17	OAD	2	Overweight, low HDL, hypertension, and one kidney (congenital renal agenesis)
P11	F	SPUPD FPG = 9.62 mmol/L HbA1c = 10.79%	25	28	Insulin	0	Overweight, nephropathy, and development delay

SPUPD, polyuropolydipsic syndrome; FPG, fasting plasma glucose; HbA1c, glycated hemoglobin; TG, triglyceride; HDL, high-density lipoprotein; LDL, low-density lipoprotein; OAD, oral anti-diabetes drug; NA, not available.

**TABLE 2:** Biochemical and immunological results of the 11 suspected MD patients.

Patient ID	FPG (mmol/L)	HbA1C (%)	Total cholesterol (mmol/L)	Triglycerides (mmol/L)	HDL (mmol/L)	LDL (mmol/L)	Creatinine (µmol/L)	CRP (mg/L)	C-peptide (ng/mL)	Pancreatic antibodies
P1	8.92	10.10	5.29	2.27	1.03	1.25	66	5.8	NA	Three negative
P2	NA	11.05	5.76	3.67	0.77	3.36	30.58	5.17	NA	Two negative
P3	20.35	8.45	5.32	0.81	1.6	3.36	42.52	4.06	0.187	Three negative
P4	19.30	12.99	NA	NA	NA	NA	NA	NA	NA	Three negative
P5	4.84	5,19	3.31	0.66	1.39	1.55	55.51	0	1.46	Three negative
P6	19.3	15.38	3.95	1.28	1.7	1.55	37	0.27	13.89	Three negative
P7	11.4	9.2	5.05	0.55	1.78	1.7	38.6	4.37	NA	Three negative
P8	15.3	8.33	3.62	0.64	1.08	2.25	71.9	5.33	0.164	Three negative
P9	10.6	7.9	3.96	0.36	2.04	1.76	57	0.77	0.068	Two negative
P10	8.24	6.5	4.64	1.32	0.8	1.2	60	2	NA	Three negative
P11	9.62	10.79	5.61	0.81	1.22	1.68	45	<5	0,4	Three negative

TC, total cholesterol; HDL, high-density lipoprotein; LDL, low-density lipoprotein; CRP, C-reactive protein; NA, not available.

C-peptide: The connecting peptide is a short 31-amino-acid polypeptide that connects insulin’s A-chain to its B-chain in the proinsulin molecule.

Three negative remains negative results for the three pancreatic antibodies.

**FIGURE 1:**
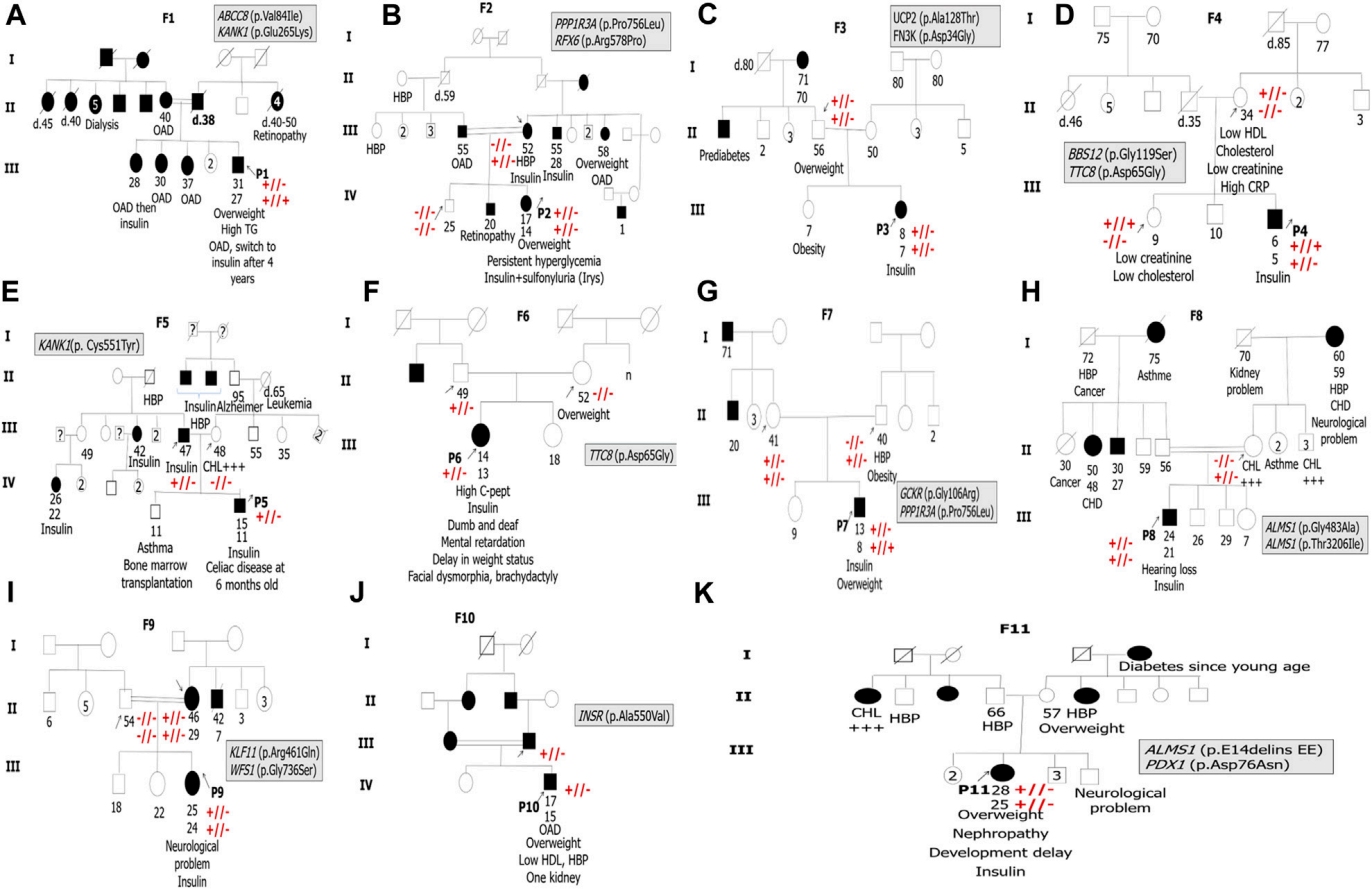
Pedigrees of the 11 suspected MD patients’ families. F indicates the proband’s family. P indicates the proband. The arrows indicate family members, whose DNA samples are available in the present study. Double horizontal lines indicate inbreeding. White squares and circles indicate healthy males and females, respectively. Black squares and circles indicate males and females with diabetes, respectively. The information below family members is ordered as follows: age at examination, age at diabetes diagnosis, clinical features, and/or specific anti-hyperglycemia treatment. OAD indicates oral antidiabetics. HBP refers to high blood pressure. CHD refers to coronary heart disease. TG refers to triglycerides. CHL +++ indicates hypercholesterolemia. Symbols + and or – indicate Sanger segregation information. +//+ indicates the presence of the variant in the homozygous state. +//- indicates the presence of the variant in the heterozygous state. -//- indicates the absence of the variant.

All patients revealed the absence of the three measured pancreatic antibodies except P2 and P9, who had positive GAD antibodies with low titer. So, immunological results confirm the eligibility of the 11 MD patients for genetic testing. The family history of diabetes and other information about clinical features are shown in Figure 1.

### Genetic findings

Our genetic investigation revealed the presence of variants in several genes, including MODY and non-MODY genes. The WES analysis among 11 suspected MD patients revealed the presence of 19 variants, of which five were novel (Table 3). Our results showed the presence of variants in a single gene for five patients, namely, P5 (*KANK1*; c.1652G>A [p.Cys551Tyr]), P6 (*TTC8*; c.194A>G [p.Asp65Gly]), P8 (*ALMS1*; c.1448G>C [p.Gly483Ala ] and c.9617C>T [p.Thr3206Ile]), P9 (*WFS1*;c.2206G>A [p.Gly736Ser]), and P10 (*INSR*; c.1649C>T [p.Ala550Val]).

**TABLE 3:** List of the filtered genetic variants identified in the 11 suspected MD patients.

Patients	Gene	Exon	Refseq	Genetic variant	Genotype	Consequence	dbSNP	gnomAD frequency	Pathogenicity score	ClinVar ID	References	ACMG classification	Final pathogenicity assessment
P1	*ABCC8* *KANK1*	2 7	NM_000352.6 NM_001,256,876.2	c.250G>A c.793G>A	Het Hom	p.Val84Ile p.Glu265Lys	- -	1.989e-05 7.571e-05	8 11	554,707 1,353,906	ClinVar ClinVar	VUS VUS	Pathogenic Likely to be Pathogenic
P2	*PPP1R3*A *RFX6*	4 16	NM_002711 NM_173560	c.2267C>T c.1733G>C	Het Het	p.Pro756Leu p.Arg578Pro	rs151310594 rs146115506	0.0009 0.0023	11 10	393,402 436,531	Clinvar ClinVar	Likely benign VUS	Likely benign Likely to be pathogenic
P3	*UCP2* *FN3K*	5 1	NM_003355 NM_022158	c.382G>A c.101A>G	Het Het	p.Ala128Thr p.Asp34Gly	rs774701085 rs368552367	8.071e-06 0.0002	12 9	- -	The present study The present study	Likely benign Likely benign	Benign Benign
P4	*BBS12* *TTC8*	2 3	NM_152618 NM_144596	c.355G>A c.194A>G	Hom Het	p.Gly119Ser p.Asp65Gly	rs77731085 rs114557412	0.0016 0.0009	9 11	215,543 262,515	LOVD LOVD	Benign likely benign	Likely to be pathogenic Likely to be pathogenic
P5	*KANK1*	3	NM_015158	c.1652G>A	Het	p.Cys551Tyr	rs370250575	0.000125	12	426,868	The present study	Benign	Likely to be pathogenic
P6	*TTC8*	3	NM_144596	c.194A>G	Het	p.Asp65Gly	rs114557412	0.0009	10	262,515	LOVD	Likely benign	Likely to be pathogenic
P7	*GCKR* *PPP1R3A*	4 4	NM_001486 NM_002711	c.316G>A c.2267C>T	Het Hom	p.Gly106Arg p.Pro756Leu	rs745900416 rs151310594	3.978e-06 0.0009	10 11	- 393,402	The present study LOVD	VUS likely benign	Likely to be pathogenic Benign
P8	*ALMS1* *ALMS1*	8 11	NM_015120	c.1448G>C c.9617C>T	Het Het	p.Gly483Ala p.Thr3206Ile	rs1462413512 rs201624771	0.0000121 0.0003	4 8	1903933 449,932	The present study The present study	Likely benign Likely benign	Likely to be pathogenic Likely to be pathogenic
P9	*KLF11* *WFS1*	4 8	NM_003597 NM_001145853	c.1382G>A c.2206G>A	Het Het	p.Arg461Gln p.Gly736Ser	rs199770737 rs71532864	0.0005 3.254e-05	10 13	2167243 1328696	LOVD LOVD	Benign pathogenic	Benign Pathogenic
P10	*INSR*	8	NM_000208	c.1649C>T	Het	p.Ala550Val	_	_	9	-	The present study	VUS	Likely to be pathogenic
P11	*ALMS1* *PDX1*	1 1	NM_015120 NM_000209	c.41_42insGGA c.226G>A	Het Het	p.E14delins EE p.Asp76Asn	rs55889738 rs137852783	_ 0.0032	_ 8	- 8859	The present study LOVD/Uniprot	Benign Benign	Benign Likely to be pathogenic

Het, heterozygous state; Hom, homozygous state; VUS, variant with uncertain significance.

Italic value indicates the names of genes.

The six remaining patients carried variants in more than one gene, such as the P1 (*ABCC8* and *KANK1*), P2 (*PPP1R3A* and *RFX6*), P3 (*UCP2* and *FN3K*), P4 (*BBS12* and *TTC8*), P7 (*PPP1R3A* and *GCKR*), and P11 (*PDX1* and *ALMS1*).

WES analysis performed in P1 led to the identification of two missense variants in *ABCC8* (c.250G>A, p.Val84Ile) and *KANK1* (c.793G>A, p.Glu265Lys) genes. According to the ACMG classification, the heterozygous *ABCC8* variant is considered as a variant of uncertain significance (VUS). Both variants are reported as VUS in ClinVar (ID:554707 and ID:1353906). Moreover, the two identified variants are very rare (gnomAD frequency = 1.989e-05 and 7.57 × 10^−5^, respectively).

Another variant in the *KANK1* gene (c.1652G>A, p.Cys551Tyr) was found in the heterozygous state in P5. The frequency of this variant is rare worldwide (gnomAD, frequency = 1.25 × 10^−4^). According to the ACMG guidelines, it is predicted as benign, although there are conflicting interpretations reported in ClinVar, classifying it as a VUS (ID: 426868).

Genetic investigation of P2 allowed the identification of two non-synonymous heterozygous variants in *PPP1R3A* (c.2267C>T, p.Pro756Leu) and *RFX6* (c.1733G>C, p.Arg578Pro) genes. The rs151310594 (p.Pro756Leu) variant located in the *PPP13A* gene is rare according to gnomAD (frequency = 0.0009). It is reported in ClinVar (ID: 393402) as associated with monogenic diabetes, while it is classified as likely benign according to the ACMG classification. (Richards et al., 2015). The second variant, c.1733G>C (p.Arg578Pro), located in the *RFX6* gene has conflicting interpretations of pathogenicity (VUS). It is a rare variant according to the gnomAD database (frequency = 2.1 × 10^−4^).

Two novel heterozygous potential pathogenic variants in *UCP2* (c.382G>A, p.Ala128Thr) and *FN3K* (c.101A>G, p.Asp34Gly) genes have been identified in P3. The two identified variants in *UCP2* and *FN3K* genes are reported for the first time, considered as likely benign according to the ACMG guidelines.

The patient P4 carries two potential pathogenic variants in *BBS12* (c.355G>A, p.Gly119Ser) and *TTC8* (c.194A>G, p.Asp65Gly) genes. First, a homozygous variant was found in the *BBS12* gene, which is considered as benign according to the ACMG guidelines, despite the conflicting interpretations reported in ClinVar (ID:215543). Second, a heterozygous rare variant (frequency = 3.23 × 10^−3^) in the *TTC8* gene (p.Asp65Gly) was found in the proband. According to the ACMG guidelines, this variant is predicted as likely benign; however, conflicting interpretations were reported in ClinVar (ID: 262515).

The variant (c.194A>G, p.Asp65Gly) identified in the *TTC8* gene was also found in the heterozygous state in P6.

The filtering and prioritization of variants conducted in P7 allowed the identification of two potential pathogenic variants. The first variant (c.2267C>T, p.Pro756Leu) identified in the *PPP1R3A* gene*,* the same found in P2, was present in both the proband and the healthy parents. This result excludes the possible pathogenic effect of this variant in this family. The second variant (c.316G>A, p.Gly106Arg) was identified in the *GCKR* gene (glucokinase regulatory protein) described for the first time (frequency = 1.97 × 10-5) and classified as a VUS in accordance with the ACMG guidelines.

The genetic results of patient P8 showed the presence of two missense variants in the *ALMS1* gene. These two variations (c.1448G>C, p.Gly483Ala and c.9617C>T, p.Thr3206Ile) are rare according to the gnomAD database (f = 0.0000121 and 0.0003, respectively). According to the ACMG classification, they are considered as likely benign. In ClinVar (IDs: 1903933 and 449932), the available evidence is currently insufficient to determine the role of these two variants in the disease. Therefore, they have been classified as VUS.

We identified another novel heterozygous variant (c.41_42insGGA) in the *ALMS1* gene for P11. We excluded this variant as it is classified as benign in line with the ACMG guidelines and ClinVar. Our genetic analysis showed the presence of another heterozygous variant in the *PDX1* gene (pancreatic and duodenal homeobox 1) in P11. It is a rare variant with conflicting interpretations in ClinVar (ID: 8859). This variant (c.226G>A, p.Asp76Asn) is predicted to be pathogenic according to the UniProt database.

Patient P9 carried two potential pathogenic variants. The first variant was identified in the *KLF11* gene. It is a rare missense variant (c.1382G>A, p.Arg461Gln) classified as benign in compliance with the ACMG guidelines and ClinVar (ID: 2167243). Therefore, we excluded this variant. The second variant (c.2206G>A, p.Gly736Ser), located in exon 8 of the *WFS1* (Wolframin ER transmembrane glycoprotein) gene, is classified as pathogenic according to the ACMG criteria.

Our genomic investigation allowed the identification of a novel heterozygous pathogenic variant (c.1649C>T, p.Ala550Val) in exon 8 of the *INSR* gene in patient P10 and in his diabetic father having type A insulin resistance. P10 is a 17-year-old male having diabetes, hypertension, overweight, low HDL-CHL, and renal agenesis.

ORVAL results are summarized in Table 4, showing the possible pathogenic combinations between the variants identified among these patients.

**TABLE 4:** Potential pathogenic variant combinations and their effect on disease-causing among suspected MD patients.

Patient ID	Variant combinations	VarCoPP score	Predicted pathogenicity	Type of digenic effect
P1	*ABCC8*:11:17496473:C:T *KANK1*: 9:711559:G:A	0.86	Disease-causing with 99% confidence	True digenic
P2	*RFX6*: 6:117246670:G:C *PP1R3A*:7:113518880:G:A	0.59	Low pathogenicity	Monogenic + modifier
P3	*FN3K*: 17:80693613:A:G *UCP2*: 11:73688018:C:T	0.52	Low pathogenicity	Dual molecular diagnosis
P4	*TTC8* : 14:89305845:A:G *BBS12*: 4:123663402:G:A	0.93	Disease-causing with 99.9% confidence	True digenic
P7	*GCKR*: 2:27721152:G:A *PP1R3A*:7:113518880:G:A	0.53	Disease-causing	True digenic
P11	No variant combination	-	-	-

Sanger sequencing results confirmed the presence of all variants identified by WES in all probands and their family members if they were available (Figure 1) (see Supplementary Table S2).

### Structural analysis

In the present study, we carried out modeling structure protein for the following patients: P5, who carried a variant in the KANK1 protein; P7, who harbored a novel variant in the GCKR protein; P8, who had two variants in the ALMS1 protein; and P10, who carried a novel variant in the INSR protein.

The predicted 3D protein structure of KANK1 (aa:180-626) was evaluated to assess the reliability of the generated model. The Ramachandran plot shows that 92.1% of the residues reside within a favorable region and 5.2% are in the allowed region. ProSA indicates a Z-score value of −4.82, which is within the Z-score range of the experimentally determined protein structure using X-ray spectroscopy (see Supplementary Figure S1A). The *KANK1* variant (c.1652G>A) induced the change of cysteine residue with a bigger and less hydrophobic tyrosine residue at position 551 of the KANK1 protein (p.Cys551Tyr). mCSM showed a decrease in KANK1 protein stability (0.732 kcal/mol). Also, DUET confirmed this finding, revealing a negative ΔΔG value of −0.561 kcal/mol. In fact, the mutant residue Tyr551 disrupts a hydrogen bond with the residue Glu554 and creates two hydrophobic interactions with Val547, as well as two polar interactions with Glu554 via its aromatic ring. It also creates two polar interactions with Gly548 (see Supplementary Figure S2B).

Regarding the *GCKR* variant (c.316G>A, p.Gly106Arg) identified in P7, it leads to the substitution of a neutral residue by a larger, hydrophobic, and positively charged residue located in the sugar isomerase domain −1 (SIS-1). mCSM and DUET showed a decrease in the protein stability, indicating a negative ΔΔG value of −0.949 kcal/mol and −0.988 kcal/mol, respectively. The 3D structure analysis showed that this substitution induced a steric clash, which may destabilize the folding of the fructose-binding domain (see Supplementary Figure S2A).

To study the effect of variants (c.1448G>C, p.Gly483Ala and c.9617C>T, p.Thr3206Ile) identified in the *ALMS1* gene in patient P8, we generated two models of the ALMS1 protein (aa: 322-521 and aa: 3162-3331) using the I-TASSER server. These models were evaluated to ensure their reliability. The Ramachandran plot analyses for the first model indicated that 91.4% of its residues were in the most favored region, while 7.6% of residues were in the allowed region (see Supplementary Figure S1B). In the second model, out of 167 amino acids, 142 (85%) were in the favored region, and 19 (11.4%) were in the generously allowed region (see Supplementary Figure S1C). Both ALMS1 models generated were within the Z-score range of the experimentally determined protein structure using X-ray spectroscopy and nuclear magnetic resonance of −1.23 and −1.26, respectively (see Supplementary Figures S1B, C). The first identified variant, p.Gly483Ala, resulted in a decrease in protein stability. Indeed, DUET displayed a slight negative ΔΔG value (−0.033 kcal/mol) as well as mCSM (−0.271 kcal/mol). It induced the loss of a hydrogen bond with Lys486 and three polar interactions with Ala485 and Lys486. Conversely, it created an additional hydrogen bond with Ser480 and two polar interactions with Ser480 (see Supplementary Figure S2D). The second variant, p.Thr3206Ile, was found to be stabilizing according to DUET (0.314 kcal/mol), while mCSM and Dynamut2 indicated a destabilizing effect (−0.221 kcal/mol and −0.45 kcal/mol respectively). This led to the loss of three hydrogen bonds with Gln3203 and Lys3269 and the appearance of two additional polar interactions with Lys3211 (see Supplementary Figure S2E).

Concerning the variant found in the *INSR* gene (c.1649C>T, p.Ala550Val) in P10, it leads to the substitution of an alanine residue with a larger residue at position 550 of the INSR protein (p.Ala550Val). This variant resulted in a decrease in protein stability. mCSM analysis displayed a ΔΔG value of −0.188 kcal/mol. However, DUET had a stabilizing effect (0.093 kcal/mol). Due to this discord, we turned to Dynamut2 and SDM, which in turn demonstrated a loss in stability by displaying negative ΔΔG values of −0.51 kcal/mol and −0.21 kcal/mol, respectively. Moreover, this variant occurs in fibronectin type III domain (FnIII-1), which is implicated in insulin biding. Comparing the 3D structure of the wild type to the mutant protein, we observed that this substitution induces the appearance of two hydrophobic interactions with Phe541 (see Supplementary Figure S2C).

### Monogenic diabetes subclasses

Both clinical traits and genetic variants helped identify diabetes subtypes among the 11 recruited patients. We noted the presence of many types of diabetes, including the idiopathic type 1 diabetes identified for P3 and MODY subclasses described for P1 (ABCC8_MODY), P2 (RFX6_MODY), P7 (GCK_MODY), and P11 (PDX1_MODY). Four patients had syndromic forms of diabetes, including the Bardet–Biedl syndrome in P4 and P6, the Alström syndrome in P8, and the insulin resistance syndrome type A in P10. Patient P9 showed isolated diabetes with significantly reduced penetrance for Wolfram syndrome-related features. An unclassified form of diabetes caused by mutation in the *KANK1* gene was identified in P5 (Table 5).

**TABLE 5:** Diabetes subtypes among the 11 Tunisian diabetic patients.

Patient ID	Typical clinical features	Causative genes	MD subtype
P1	Fasting hyperglycemia, overweight, and hypertriglyceridemia	*ABCC8* and *KANK1*	MODY_ 12 (ABCC8)
P2	Diabetes, abdominal pain, and vomiting	*RFX6*	RFX6_MODY
P3	Ketoacidosis and obesity	*UCP2* and *FN3K*	Idiopathic type 1 diabetes
P4	Diabetes only	*BBS12* and *TTC8*	Bardet–Biedl syndrome
P5	Diabetes	*KANK1*	Monogenic diabetes
P6	Dumbness and deafness, mental retardation, delay in weight status, neurological problems, facial dysmorphia, and brachydactyly	*TTC8*	Bardet–Biedl syndrome 8
P7	No ketoacidosis, nervous character, learning difficulties, and overweight	*GCKR*	MODY_2 (GCK_MODY)
P8	Hearing loss, retinitis pigmentosa, and neurological problem	*ALMS1*	Alström syndrome
P9	Diabetes and neurological problems	*WFS1*	Isolated diabetes with low penetrance for Wolfram syndrome features
P10	Overweight, kidney agenesis, low HDL, and hypertension	*INSR*	Insulin resistance type A
P11	Nephropathy, development delay, and overweight	*ALMS1* and *PDX1*	MODY_4 (PDX1_MODY)

Italic value indicates the names of genes.

These genetic findings were taken into account and were communicated to the referring physicians in order to adjust the treatment for better healthcare management of the diabetic patients. Among the 11 studied patients, genetic analysis allowed precise medication adjustment for three patients.

## Discussion

MD is a health problem with major health complications in certain admixed populations, such as the Tunisian population (Kefi et al., 2015), which is characterized by a high level of consanguinity, which increases the occurrence of genetic disorders (Ben Halim et al., 2013). In this study, 11 suspected MD patients were screened for MD using WES. We observed four cases of MODY subtypes, four cases with syndromic forms of diabetes, one patient with idiopathic T1D, one patient with an isolated form of diabetes with reduced penetrance or non-penetrance for other Wolfram syndrome-related features, and one unclassified case with an unknown form of diabetes. Our genomic investigation pinpoints the high clinical and genetic heterogeneity of MD among Tunisians.

According to the OMIM database, mutations in the *ABCC8* gene are associated with maturity-onset diabetes of the young (MODY_12, MIM **#** 125853). The identified variant (p.Val84Ile) in P1 is located in a conserved region of transmembrane domain 0″ TMD0” of sulfonylurea receptor 1 (SUR1) and causes non-neonatal diabetes mellitus (Meng Li et al., 2021). Our result is in accordance with another study showing the wide spectrum of clinical forms of *ABCC8* mutations, ranging from permanent neonatal diabetes mellitus (PNDM) to less severe forms of diabetes with variable expression and age at onset (M. Li, Han, and Ji, 2021). The identified variant was reported first in a European ancestry man who developed diabetes at an early age (12 years old) with persistent mild hyperglycemia (Gonsorcikova et al., 2011), unlike our index case, P1, who developed diabetes at the age of 27 with a high fasting hyperglycemia (19.08 mmol/L). P1 clinical profile bears a resemblance to a previously reported Tunisian patient harboring a variant in the same gene (ABBC8_MODY) (Dallali et al., 2019).

The clinical severity observed in this patient could be explained by the presence of another variant in the *KANK1* gene, claimed to be associated with FPG in East Asians (Hwang et al., 2015). Actually, the KANK1 protein (KN motif and ankyrin repeat domain-containing protein 1) plays a role in cytoskeleton formation by regulating actin polymerization and negatively regulating Rac1 and RhoA G protein signaling pathways that have been implicated in insulin secretion (Chundru et al., 2021). ORVAL tool interrogation confirmed the potential pathogenic effect of the *ABCC8–KANK1* combination with 99% confidence. It seems that these two variants interact together with a true digenic disease-causing mechanism. In other words, the coexistence of these two variants is at the fundamental cause of disease etiology in this patient. To sum up, these arguments suggest the combined causative effect of the identified variants within the *ABCC8* and *KANK1* genes and confirm the genotype/phenotype correlation in P1. Our genetic testing prompted the referring physician to recommend P1 to stop insulin and switch to the combination of sulfonamides with glinides. This precise medication adjustment seems to be much more efficient for better glucose homeostasis of P1 (Ovsyannikova et al., 2016). All of P1’s family members developed diabetes before the age of 40. Unfortunately, we do not have their samples to search for the proband’s variants and provide for genetic counseling.

**FIGURE 2:**
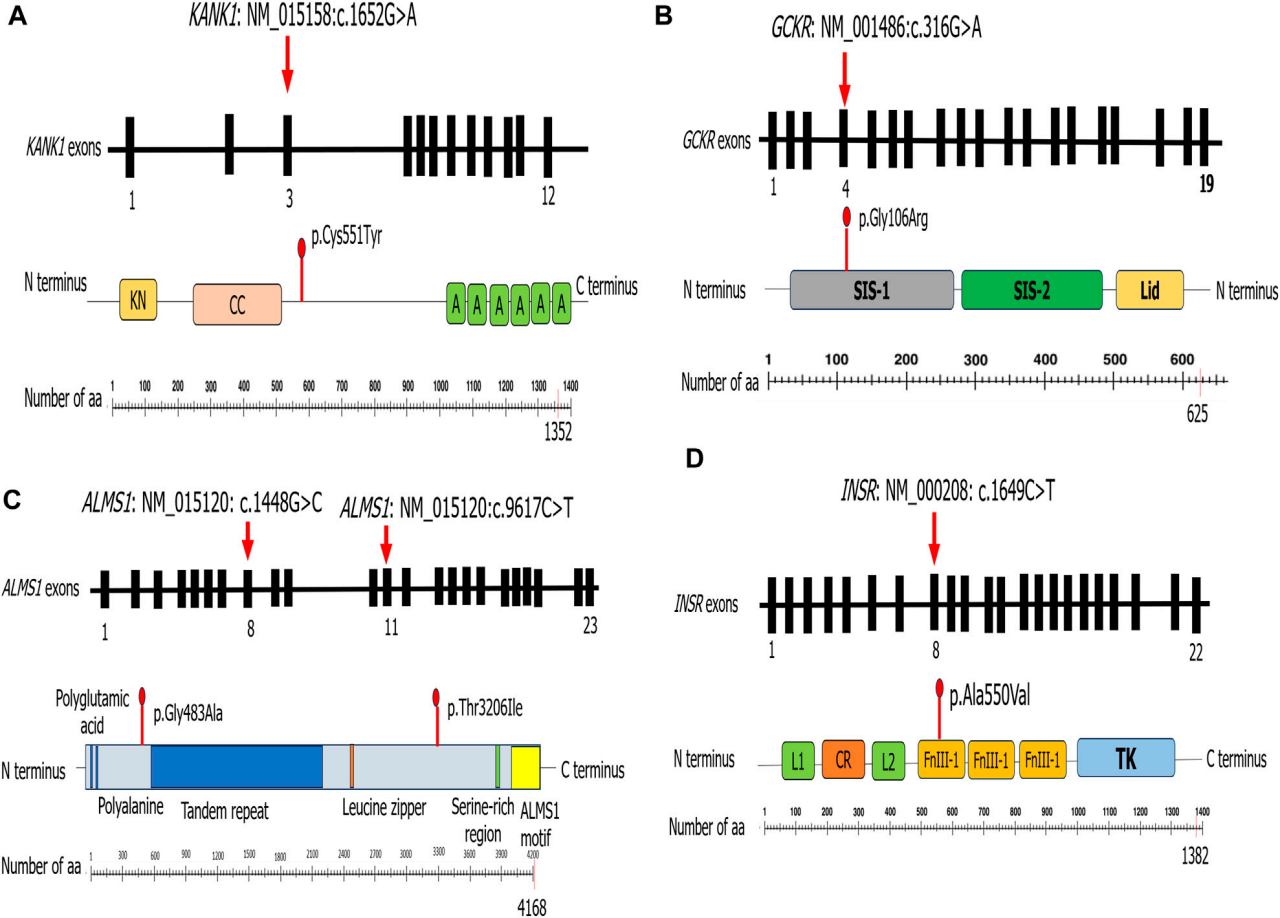
Schematic representation of the different proteins for which we performed structural modeling.

Patient P5 had celiac disease since birth, which led us to suspect T1D, but the absence of anti-pancreatic autoantibodies excluded this suggestion. WES identified a variant (p.Cys551Tyr) in *KANK1* that results in a non-conservative amino acid substitution, which likely impacts the secondary structure of the protein as these residues differ in polarity, charge, size, and/or other properties. Advanced modeling of the protein sequence and biophysical properties (such as structural, functional, and spatial information and amino acid conservation, physicochemical variation, residue mobility, and thermodynamic stability), as well as our structural analysis (Figure 2A) (see Supplementary Figure S2B), indicates that this missense variant is expected to disturb KANK1 protein function. In addition, Sanger sequencing confirmed the presence of the KANK1 variant in both the patient and his diabetic father. This variant was absent in the healthy mother. Taking into account all these arguments, this variant is likely to be pathogenic. It is important to perform *in vivo* functional studies to confirm the true impact of this variation on the onset of diabetes.

The *PPP1R3A* gene (the protein phosphatase 1 regulatory subunit 3) encodes the regulatory subunit that binds to muscle glycogen with high affinity, thus playing an important role in glycogen metabolism. This gene is associated with T2D and lipid metabolism (Zhang et al., 2014). In the literature, a total of 15 variants have been identified in the *PPP1R3A* gene at present (Doney et al., 2003). In our study, the rs151310594 identified in the *PPP1R3A* gene was absent in the diabetic mother, thus ruling out the possibility of its role in the development of diabetes in this family. The second variant, c.1733G>C (p.Arg578Pro) located in the *RFX6* gene, was identified in seven individuals from 2,793 Indian people, which seems to be linked to monogenic diabetes (Narang et al., 2020). The *RFX6* gene regulates the transcription factors involved in beta-cell maturation and function (Wurdeman et al., 2017). It was reported to be associated with the Mitchell–Riley syndrome (MRS, MIM**#** 615710), which is characterized by neonatal diabetes with pancreatic hypoplasia, duodenal and jejunal atresia, and gall bladder agenesis (Zegre Amorim, 2015). Until now, only eight patients have been confirmed with MRS presenting all the typical clinical traits, contrary to our patient, who presented atypical clinical features of MRS characterized by abdominal pain and vomiting. All reported cases of MRS were found to have homozygous or compound heterozygous variants in *RFX6* (Reh, 2014), in opposition to our index P2, who carries the RFX6 variant in the heterozygous state. In addition to that, the *RFX6* variant was also present in the mother without any typical clinical signs of MRS. All these observations refute the diagnosis of MRS in this family; however, according to the literature, individuals with *RFX6* heterozygous variants may be more susceptible to develop type 2 diabetes at a younger age (Sansbury et al., 2015). In this context, Patel et al. showed that heterozygous variants in *RFX6* can cause mild MODY with reduced penetrance (Piccand et al., 2014). In fact, patients with RFX6-MODY had normal development of the *β* islet cells but defective insulin secretion, leading to hyperglycemia (Homeostasis et al., 2014). In our study, the *RFX6* variant was confirmed in the patient and her mother, who had diabetes since the age of 31 years. ORVAL output revealed a low VarCopp score of 0.59, indicating the low possibility of interaction between these two variants. In all, we retained only the RFX6 variation describing the RFX6_MODY as the likely cause of disease subtype in this family. Our results emphasize the need for a deeper investigation into the function of the *RFX6* gene to pave the way for novel therapeutic approaches (Passone et al., 2022).

*UCP2* is a protein-coding gene of the mitochondrial uncoupling protein (UCP) that plays a role as a critical link between obesity, cell dysfunction, and type 2 diabetes (J. Li et al., 2019). Concerning the *FN3K* gene, it encodes an enzyme that catalyzes the phosphorylation of fructosamines, which may result in deglycation. Among its related pathways are the metabolism of proteins and Gamma carboxylation. Genome-wide association studies (GWAS) revealed a link between *FN3K* variants and elevating hemoglobin A1 (HbA1C) measurement and the onset of type 2 diabetes and its complications (Dunmore et al., 2018). The presence of both variants among the proband and the unaffected father does not explain the occurrence of diabetes but rather the genetic predisposition to obesity in this family. A low pathogenicity of variant combination has been detected by ORVAL (low VarCopp score). On the other hand, the presence of diabetic ketoacidosis, the onset of hyperglycemia, the extremely low C-peptide (0.187 ng/mL), and the absence of the three pancreatic antibodies fulfilled the diagnostic criteria of the idiopathic T1D (Kaneko et al., 2017). In addition, this atypical form of T1D has been reported since 2000 as a novel subtype of T1D characterized by the presence of a combination of typical signs of T1D (diabetic ketoacidosis) and clinical course that often resembles T2D. Moreover, idiopathic T1D is most commonly seen in obese African–American individuals (Sobngwi and Gautier, 2002). More interestingly, 4% of a cohort of children with monogenic diabetes in Qatar had idiopathic T1D (Abdel-Karim et al., 2021). Based on our clinical and genomic findings, the proband was assigned a diagnosis of idiopathic T1D, and she is predisposed to obesity; thus, nutritional follow-up with a hypocaloric diet has been highly recommended for early disease prevention.

According to the literature, the *BBS12* gene is associated with Bardet–Biedl syndrome (BBS), an autosomal recessive genetic disorder characterized by the presence of the following primary features: retinal degeneration, central obesity, postaxial polydactyly, learning problems, and renal abnormalities. Moreover, BBS is often complicated by other minor symptoms, including hepatic fibrosis, diabetes mellitus, neurological problems, speech and lingual deficits, facial dysmorphism, dental anomalies, developmental delay, hypertension, brachydactyly/syndactyly, cardiovascular anomalies, reproductive anomalies, short stature, and hearing loss (Khan et al., 2016) (MIM # 615989). Our results confirm, in part, the findings of Deveault et al., who identified the same variant in the homozygous state in a patient fulfilling the BBS criteria (Deveault et al., 2011). Our patient presents some minor clinical criteria of BBS, including behavioral problems, learning disabilities, and short stature. Many European ancestry patients carrying the same variant were diagnosed with BBS in the absence of major clinical characteristics (Billingsley et al., 2010; Chen et al., 2011). This clinical heterogeneity may be explained by the high genetic specificity and signature of BBS among populations even with the late onset of clinical signs. Another heterozygous variant in the *TTC8* gene (p.Asp65Gly) was found in the proband. Indeed, the *TTC8* (tetratricopeptide repeat domain 8) is a protein-coding gene expressed in ciliated cells involved in the formation of cilia. This gene is directly linked to BBS (Sato et al., 2019). In fact, the *TTC8* variant (p.Asp65Gly) led to the change of an aspartic acid amino acid to glycine (p.Asp65Gly), which is a hydrophobic amino acid. Thus, it may have an impact on the protein function. This variant was absent in the unaffected mother and sister. The ORVAL outputs a high VarCopp score = 0.93, showing a true digenic combination between the two variants. This result confirmed well the absence of diabetes among the mother and the sister, who carried only one mutation. Our results are in line with the finding of Dallali et al., who identified an oligogenic inheritance of BBS in a Tunisian patient (Dallali et al., 2021). This case highlighted the clinical variability in BBS, which may be underdiagnosed in patients with milder phenotypes.

The variant (c.194A>G, p.Asp65Gly) identified in the *TTC8* gene was also found in the heterozygous state in P6. Sanger sequencing confirmed the presence of this variant in both the index and the father. The father presented minor clinical features of BBS, including distinctive facial dysmorphia, dental abnormalities, and short stature. The proband presented both major BBS clinical features, namely, learning difficulties, and minor BBS traits such as speech deficits, development delay, diabetes mellitus, and brachydactyly. This case draws attention to the high clinical heterogeneity of BBS not only at the population level but also at the family level. Further genetic investigations, such as long-read sequencing, will be useful in order to identify structural variations that were missed in the WES analysis. We recommend screening for other BBS clinical features in this family.

The *GCKR* gene encodes a regulatory protein that inhibits glucokinase (GCK) enzyme in the liver and pancreatic islet cells. The effect of the GCKR protein depends on the presence of fructose 6-phosphate and is antagonized by fructose 1-phosphate. Given the role of glucokinase in the development of maturity-onset diabetes of the young GCK_MODY or MODY_2 (MIM # 125851), mutations in GCKR have been considered to be associated with MODY_2 (The et al., 2001). A recent study showed an association between GCKR mutations and high FPG levels, triglyceride measurement, and obesity (Langer et al., 2021). Previous studies reported that a common genetic variant in *GCKR* (rs1260326) was associated with varying metabolic characteristics (Goodman et al., 2020; Zahedi et al., 2021). Until today, only four variants in the *GCKR* have been reported as associated with monogenic disorders. The 3D structure analysis of the GCKR protein showed that p.Gly106Arg is located in sugar isomerase domain −1 (SIS-1) (Figure 2B) and induced a steric clash, which may destabilize the folding of the fructose-binding domain (see Supplementary Figure S2A). Sanger sequencing showed the presence of this variant in the obese father and its absence in the healthy mother. All these scientific data affirm the involvement of this variant in the appearance of diabetes and obesity in the index case. In addition, ORVAL analysis excludes the interaction between *PPP1R3A* and *GCKR* genes (low VarCoPP score = 0.53). Based on the genetic findings, the treatment was adjusted from high doses of insulin to low doses of insulin associated with a healthy lifestyle, as recommended by a previous study (Anik et al., 2015). Interestingly, this variant (c.316G>A; p.Gly106Arg) is associated with diabetes in P7 and with obesity in his father. Hence, we highlighted the clinical variability of this variant in the same family. To the best of our knowledge, we are the first to describe *GCKR* variation in a Tunisian MD patient.

Homozygous or compound heterozygous variant mutations in the *ALMS1* gene may cause Alström syndrome (MIM#606844), characterized by a progressive loss of vision and hearing, heart disease, obesity, T2D, and short stature. This disorder can also cause serious problems involving the liver, kidneys, bladder, and lungs. In this study, P8 had hearing loss, diabetes, and neurological problems, which suggest the diagnosis of Alström syndrome. In line with a previous study showing that approximately 70% of patients during infancy or adolescence with Alström syndrome develop cardiomyopathy (Dassie et al., 2021), P8 may develop a heart condition with age. Similarly, Hearn et al. reported a patient with Alström syndrome carrying compound heterozygous variant mutations in the *ALMS1* gene (Hearn et al., 2002). Our 3D structural analysis revealed that the first variant (c.1448G>C, p.Gly483Ala) (Figure 2C) induces the loss of a hydrogen bond with Lys486 and three polar interactions with Ala485 and Lys486. Conversely, it creates an additional hydrogen bond with Ser480 and two polar interactions with Ser480 (see Supplementary Figure S2D). The second variant (c.9617C>T, p.Thr3206Ile) (Figure 2C) leads to the loss of three hydrogen bonds with Gln3203 and Lys3269 and the appearance of two additional polar interactions with Lys3211 (see Supplementary Figure S2E). Sanger sequencing confirmed the presence of the two *ALMS1* variations in the proband. The healthy mother carried only one variant mutation (c.9617C>T) in the heterozygous state. Taking into account all these findings, we confirmed the diagnosis of Alström syndrome in P8. Functional analysis is needed in order to better understand the effect of these two variants.

*PDX1* is associated with the MODY_4 subtype (MIM#600733). Reading through the literature, MODY_4 or *PDX1*_MODY is a rare form of monogenic diabetes caused by heterozygous variants in *PDX1* that leads to pancreatic beta-cell dysfunction. Up to date, only few mutations in the *PDX1* gene have been linked to monogenic diabetes. In 2015, three new patients with MODY were described as harboring variants in *PDX1* (Mangrum et al., 2015). At a clinical level, our patient has diabetes and is overweight. This result corroborates a previous study highlighting the diagnosis of MODY_4 in obese and non-obese patients with hyperglycemia (Abreu et al., 2021). Regarding medication, MODY_4 patients have a variable treatment response, which depends on the mutation and its consequences. In fact, some patients require only a strict diet, while others show improvement with oral diabetes medications and/or insulin (Abreu et al., 2021).

The variant (c.2206G>A, p.Gly736Ser) located in exon 8 of the *WFS1* (Wolframin ER transmembrane glycoprotein) gene was present in P9 and her diabetic mother and absent in the healthy father. According to the literature, 88% of variants located in exon 8 of the WFS1 gene are associated with WS (Hardy et al., 1999), characterized by diabetes, optic atrophy, and deafness. Our results conflict with the diagnosis of the classical form of WS for P9 and her mother because of the absence of other clinical features of WS, such as optic atrophy and deafness. Hence, the diagnosis of P9 and her mother is an isolated form of diabetes with reduced WS-related features. Our case supports other studies that have shown some *WFS1* variant mutations causing isolated diabetes with significantly reduced penetrance or non-penetrance for other WS-related features (Bonnycastle et al., 2013; Bansal, Boehm, and Darvasi, 2018). Our results are in agreement with previous studies displaying the high clinical heterogeneity of *WFS1* variants in both heterozygous (Rendtorff et al., 2011) and compound heterozygous patients (Lieber et al., 2012), giving the example of a French diabetic girl harboring the same variant (c.2206G>A, p.Gly736Ser) described in our patient P9 and showing all clinical features of WS, including diabetes, optic atrophy, and neurologic symptoms (Giuliano et al., 2005). All these arguments confirm the causative effect of this variant in the development of diabetes in this family. Our results require regular clinical monitoring of the patient and her family for optic atrophy and other manifestations of WS.

The insulin receptor (*INSR*) gene encodes a member of the receptor tyrosine kinase family, which is proteolytically processed to generate alpha and beta subunits that form a heterotetrameric receptor. The binding of insulin or other ligands to this receptor activates the insulin signaling pathway, which regulates glucose uptake and release, as well as the synthesis and storage of carbohydrates, lipids, and proteins (Ansarullah et al., 2021). Variant mutations in this gene underlie the inherited severe insulin resistance syndromes, including type A insulin resistance, Donohue syndrome (leprechaunism), and Rabson–Mendenhall syndrome (Rojek et al., 2021). The diagnosis of these disorders is difficult and complex since they share the same clinical spectrum, affecting many organ functions (kidneys, heart, skin, muscles, and others).

For our patient P10, we excluded the diagnosis of Donohue syndrome (leprechaunism) and Rabson–Mendenhall syndrome since they are typically life-threatening, contrary to the insulin resistance syndrome type A, whose symptoms frequently do not manifest until adolescence or later. Insulin resistance syndrome type A can be caused by heterozygous, homozygous, or compound heterozygous variant mutations in the *INSR* gene (MIM#147670).

In affected males, symptoms of insulin resistance syndrome type A are less obvious than in females, characterized by diabetes mellitus, insulin resistance, and acanthosis nigricans (Musso et al., 2004).

Our 3D structure analysis showed that the variant c.1649C>T, p.Ala550Val identified in P10 occurs in the fibronectin type III domain (FnIII-1), leading to the appearance of two hydrophobic interactions with Phe541 (Figure 2D). Our findings are different from those reported in the literature, showing that variant mutations in the FnIII domains cause Donohue syndrome more frequently than milder type A insulin resistance (Hosoe et al., 2021; Yu et al., 2022). Type A insulin resistance is associated with variant mutations affecting the TK domain. Indeed, variant mutations in the TK domain (β subunit) are significantly higher in patients with insulin resistance type A (59.3%) than in patients with Donohue syndrome (12.5%) (*p* = 0.00025) or patients with Rabason–Mendenhall syndrome (26.1%) (*p* = 0.024) (Hosoe et al., 2017).

We highlighted clinical heterogeneity related to *INSR* gene variations. Hence, the importance of studying more cases is highlighted, in particular from under-represented populations, such as the Tunisian population characterized by a mosaic genetic structure.

Regarding treatment, for patient P10 harboring a variant in the *INSR* gene associated with insulin resistance syndrome, medication adjustment was made from a treatment based on a combination of metformin with sulfonylureas to a treatment based only on Glucophage (Metformin^®^) (Moreira et al., 2010). This therapy has improved the patient’s glycemia.

In this work, multiple gene mutations were identified as associated with MD, which emphasizes the high clinical and genetic heterogeneity of MD among the Tunisian population. This variability may be explained by genetic, environmental, and behavioral factors.

Through this study, we highlight the relevance of an early genetic testing of MD. Providing an accurate molecular diagnosis of MD is of significant importance for both diabetic patients and clinicians for many reasons. Indeed, molecular diagnosis 1) improves MD patient classification and treatment and avoids unnecessary medication. For example, in our study, patients P1, P7, and P10 received precise medication adjustment. Also, it 2) provides insights into the prognosis and long-term management of the disease, 3) informs family screening in order to prevent complications and improve overall family health, and 4) enhances knowledge about pathophysiological mechanisms of MD to develop targeted therapies.

Taking into account all these points, molecular diagnosis of MD has a significant socio-economic impact on the public healthcare system, particularly in developing countries.

## Conclusion

MD is a collection of inherited disorders of non-autoimmune diabetes that remains insufficiently diagnosed despite increasing awareness. Proper recognition of the clinical manifestations, family history, and genetic testing are important to efficiently diagnose MD and to ensure better healthcare management of patients.

The present study highlighted the high complexity and heterogeneity of MD among Tunisian patients. Through our genetic analysis, we underline the importance of advancing human genetics in low- and middle-income countries to improve public healthcare and increase knowledge about uncommon forms of diabetes. Additional research and rigorous investigations are required to better understand the physiopathological mechanisms of MD and to implement efficient therapies that take into account genomic context and other related factors.

## Figures and Tables

**Table:** 

**aa**	Amino acid
**ACMG**	American College of Medical Genetics
**ADA**	American Diabetes Association
**BBS**	Bardet–Biedl syndrome
**CRP**	C-reactive protein
**FPG**	Fasting plasma glucose
**GAD**	Glutamic acid decarboxylase
**GnomAD**	Genome Aggregation Database
**HbA1c**	Glycated hemoglobin
**HDL**	High-density lipoprotein
**IA2**	Islet antigen 2
**ICA**	Islet cell antibodies
**IDF**	International Diabetes Federation
**LDL**	Low-density lipoprotein
**MAF**	Minor allele frequency
**MD**	Monogenic diabetes
**MDS**	Monogenic diabetes syndrome
**MENA**	Middle East and North Africa
**MIDD**	Maternally inherited diabetes and deafness
**MODY**	Maturity-onset diabetes of the young
**MRS**	Mitchell–Riley syndrome
**NGS**	Next-generation sequencing
**NMR**	Nuclear magnetic resonance
**NNDM**	Neonatal diabetes mellitus
**OAD**	Oral anti-diabetic
**ORVAL**	Oligogenic Resource for Variant AnaLysis
**PCR**	Polymerase chain reaction
**T1D**	Type 1 diabetes
**T2D**	Type 2 diabetes
**VUS**	Variant with uncertain significance
**WES**	Whole-exome sequencing
**WS**	Wolfram syndrome

## Data Availability

The datasets presented in this study can be found in online repositories. The names of the repository/repositories and accession number(s) can be found at: ClinVar repository (ID: 16876652): SCV004100827-SCV004100840.
