# Supplementary figures and images for: Whole-exome sequencing reveals novel variants of monogenic diabetes in Tunisia: impact on diagnosis and healthcare management
Source: Front Genet. 2023 Dec 14;14:1224284. doi: 10.3389/fgene.2023.1224284 (PMC10757615; doi:10.3389/fgene.2023.1224284)

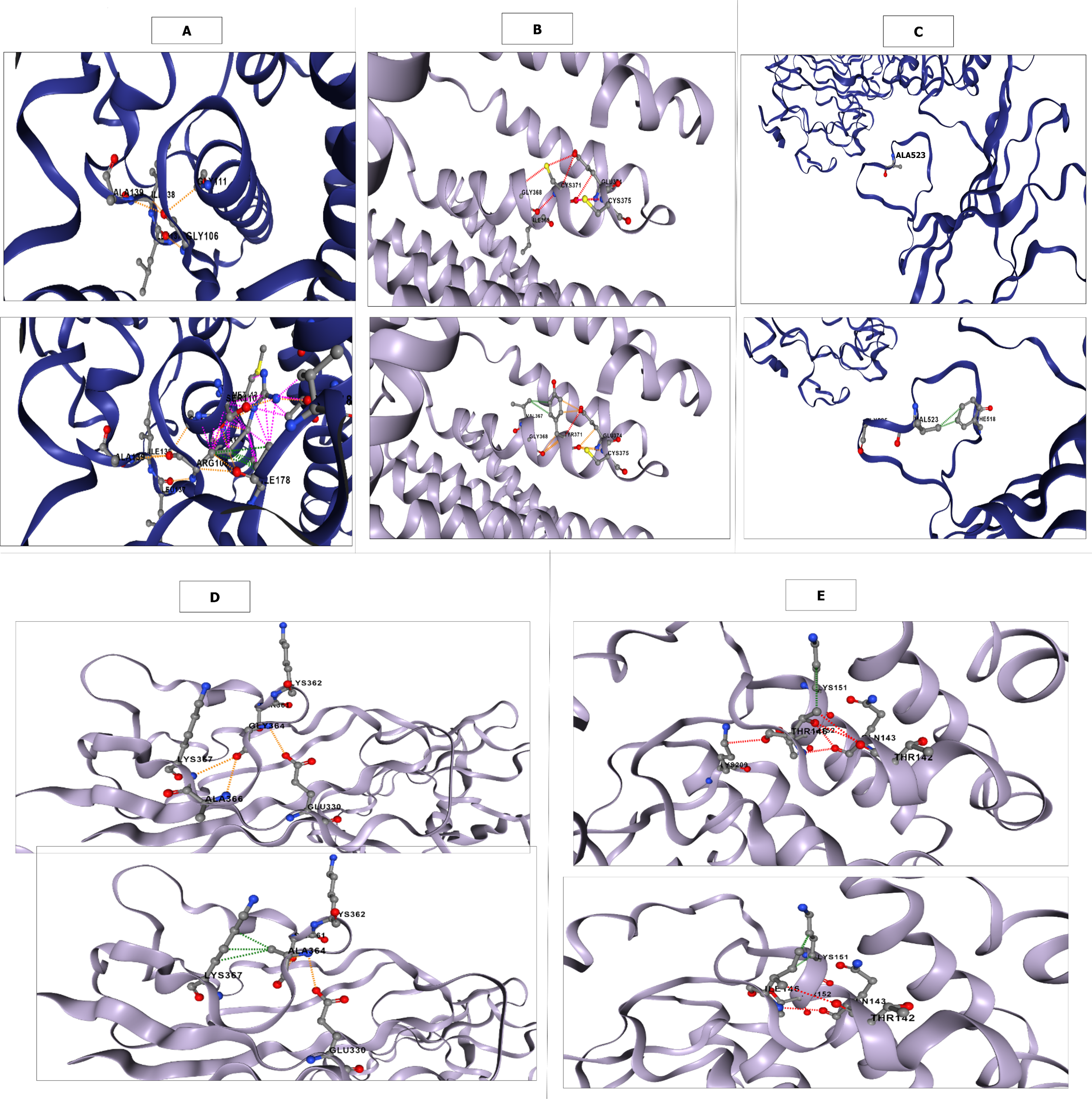

Supplement: Supplementary file 1 [file Image2.TIF]

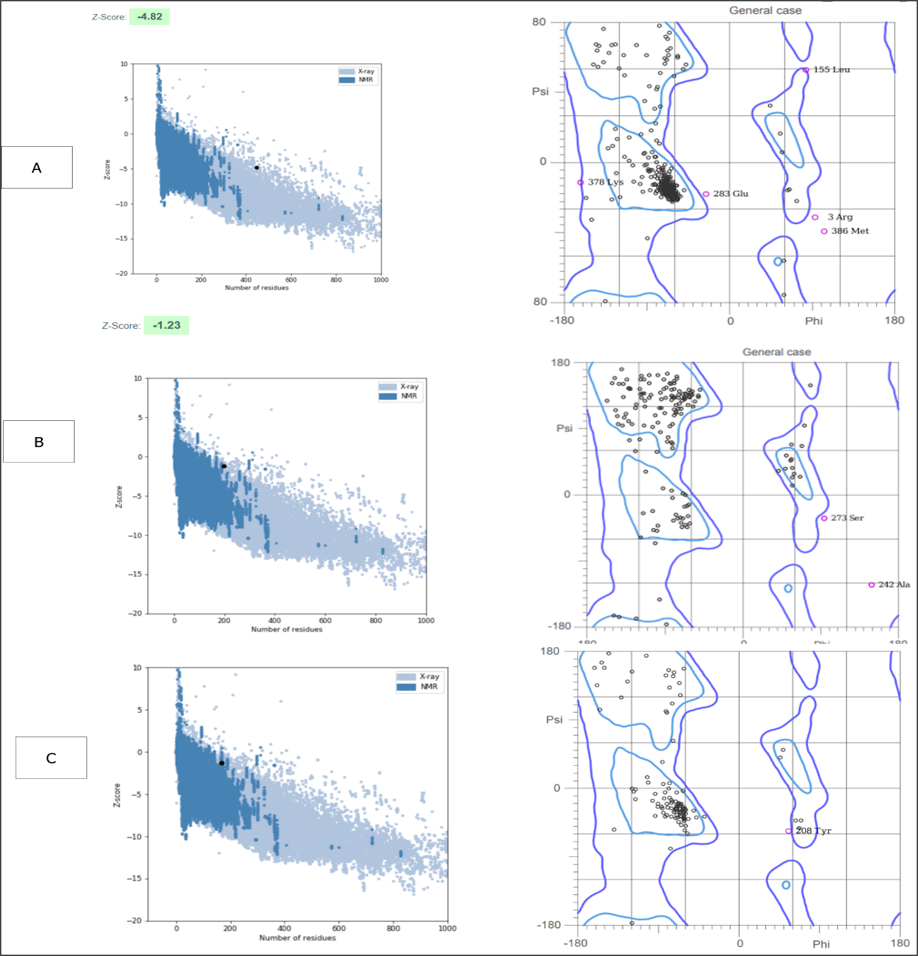

Supplement: Supplementary file 2 [file Image1.TIF]
