# Supplementary material for: Whole-exome sequencing reveals novel variants of monogenic diabetes in Tunisia: impact on diagnosis and healthcare management
Source: Front Genet. 2023 Dec 14;14:1224284. doi: 10.3389/fgene.2023.1224284 (PMC10757615; doi:10.3389/fgene.2023.1224284)
Supplement: Supplementary file 3 [file DataSheet1.pdf]

Supplementary Material

Whole Exome Sequencing reveals novel variants for monogenic diabetes in Tunisia: impact on diagnosis and health care management

Nadia Kheriji[1,2,3], Hamza Dallali[1], Ismail Gouiza[1,2,3,4], Meriem Hechmi[1], Faten Mahjoub[2,5,6], Mehdi Mrad[2,3,7], Asma Krir [7], Manel Soltani[7], Hajer Trabelsi[7], Walid Hamdi[8], Afef Bahlous[2,7], Melika Ben Ahmed[2,8], Henda Jamoussi[2,5,6], Rym Kefi*[1,2]

[1] Laboratory of Biomedical Genomics and Oncogenetics, Institut Pasteur de Tunis, BP 74, 13 Place Pasteur, 1002 Tunis, Tunisia.

[2] University of Tunis El Manar, El Manar I, 2092 Tunis, Tunisia.

[3] Faculty of Medicine of Tunis, Tunis, Tunisia

[4]University of Angers, MitoLab Team, Unité MitoVasc, UMR CNRS 6015, INSERM U1083, SFR ICAT, France
[5]Research Unit UR18ES01 on "Obesity", Faculté de Médecine de Tunis

[6] National Institute of Nutrition and Food Technology, 11 rue Jebel Lakhdar, Bab Saadoun, 1007 Tunis, Tunisia.

[7]Laboratory of Clinical Biochemistry and Hormonology, Institut Pasteur de Tunis, BP 74, 13 Place Pasteur, 1002 Tunis, Tunisia

[8] Laboratory of Clinical Immunology, Institut Pasteur de Tunis, BP 74, 13 Place Pasteur, 1002 Tunis, Tunisia.

***Correspondence**

Corresponding Author

rymkefi@pasteur.utm.tn

1. Supplementary Tables

Table S1. List of genes associated with monogenic diabetes (MD) extracted from the literature.

**Genes**	**NCBI Reference Sequence**	***reference***
ABCC8	NM_001287174.2	PMID: 33013711
ADRA2A	NM_000681.4	PubMed: 27376152
ADCY5	NM_183357.3	PMID: 34169461
AGPAT2	NM_006412.4	PMID: 11479539
AIRE	NM_000383.4	PMID: 27426947
AKT2	NM_001626.6	PubMed: 29439679
ALMS1	NM_001378454.1	PMID: 11941369

ANK1	NM_000037.4	PMID: 28912869
ARL6	NM_001278293.3	PMID: 24041679
ATM	NM_000051.4	PMID: 31814751
BCL11A	NM_022893.4	PubMed: 30250039
BCL2	NM_000633.3	PMID: 30799288
BLK	NM_001715.3	PMID: 19667185
BSCL2	NM_001122955.4	PMID: 32349771
CAV1	NM_001753.5	PubMed: 26176221
CDKN2A	NM_000077.5	PMID: 27155872
CDKN2B	NM_078487.2	PMID: 26551672
CEL	NM_001807.6	PMID: 16369531
CEP290	NM_025114.4	PMID: 24041679
CFTR	NM_000492.4	PMID: 30192635/PMID: 33500570/PMID: 33795864
CIDEC	NM_022094.3	PMID: 20049731
CISD2	NM_001008388.5	PMID: 17846994
DCAF17	NM_025000.4	PMID: 19026396
DMPK	NM_001081563.2	PMID: 31848583
EIF2AK3	NM_004836.7	PMID: 10932183/PMID: 17141632/PMID: 26231457
ERAP2	NM_001130140.2	PMID: 25201977
FAM58A	NM_152274.5	PMID: 29947923
FN3K	NM_022158.4	21288167
FOXA1	NM_004496.5	PMID: 23443045
FOXA2	NM_021784.5	28973288
FOXA3	NM_004497.3	25672906
FOXP3	NM_014009.4	30443250
FTO	NM_001080432.3	PMID: 24646999
FXN	NM_000144.5	PMID: 26704351
G6PC2	NM_021176.3	15044018
GATA4	NM_002052.5	PMID: 24330461/PMID: 26231457/ PMID: 33865372
GATA6	NM_005257.6	PMID: 28049534
GCK	NM_000162.5	PMID: 24097065
GCKR	NM_001486.4	PMID: 24226772
GIPR	NM_000164.4	PMID: 20081857/PMID: 28744963/
GLIS3	NM_001042413.2	PMID: 16715098/PMID: 29606121/PMID: 26231457

GLP1R	NM_002062.5	PMID: 30718926/PMID: 31430273/
GLUD1	NM_005271.5	PMID: 16416420
GPC6	NM_005708.5	PMID: 30511388/PMID: 22791750
GRB10	NM_001001555.3	PMID: 34234147/PMID: 28783164
GRK5	NM_005308.3	PMID: 29773828
HADH	NM_001184705.3	PMID: 27864352
HFE	NM_000410.4	PMID: 27317329
HHEX	NM_002729.5	PMID: 24736842
HIP1	NM_005338.7	PMID: 29686024/PMID: 26160912
HK1	NM_033500.2	PMID: 21781351/
HLA-B	NM_005514.8	PMID: 31843946
HMGA2	NM_003483.6	PMID: 23202124/PMID: 20581827
HNF-1A	NM_000545.8	PMID: 34035238/PMID: 30455330
HNF-1B	NM_000458.4	PMID: 28420700
HNF-4A	NM_001258355.2	PMID: 31391355
HYMAI	NR_002768.2	PMID: 34422424
IDE	NM_004969.4	PMID: 19809796
IER3IP1	NM_016097.5	PMID: 21835305/PMID: 26231457
IGF1	NM_001111283.3	PMID: 9284701/PMID: 16642022
IGF2	NM_000612.6	PMID: 24390345
IKBKAP	NM_003640.5	PMID: 28783164
IL2RA	NM_000417.3	PMID: 17676041
INS	NM_001185098.2	30414308/ 31605659
INSR	NM_000208.4	PMID: 23302862
IRS1	NM_005544.3	PMID: 19734900
ISL1	NM_002202.3	PMID: 31253982
KANK1	NM_001256876.3	PMID: 33424774/PMID: 29621232
KCNJ11	NM_000525.4	PMID: 15115830/PMID: 30169531
KCNQ1	NM_000218.3	PMID: 32695830/PMID: 23271129
KL	NM_004795.4	PMID: 27916483
KLF11	NM_003597.5	PMID: 15774581
LAMA1	NM_005559.4	PMID: 27402621
LEP	NM_000230.3	PMID: 29670283

LEPR	NM_002303.6	PMID: 29670283
LMNA	NM_170707.4	PMID: 17994215
LMNB2	NM_032737.4	PMID: 16826530
LPP	NM_001167672.3	PMID: 24509480
MADD	NM_130470.3	PMID: 24379354
MAFB	NM_005461.5	29779709
MAPK8IP1	NM_005456.4	PMID: 10700186
MC4R	NM_005912.3	PMID: 33045043
MNX1	NM_005515.4	PMID: 24411943
MTNR1B	NM_005959.5	PMID: 22286214
MYT1	NM_004535.3	33710394
NEUROD1	NM_002500.5	PMID: 10545951/ 33477506
NEUROG3	NM_020999.4	28724572
NOTCH2	NM_024408.4	PMID: 18372903/ 32312275
PAX4	NM_006193.2	15842522/ 21263211/22296034
PAX6	NM_001258462.3	33782094
PCNT	NM_006031.6	PMID: 32671003
PCSK1	NM_000439.5	24890885
PDX1	NM_000209.4	PMID: 30930126/33477506
PEPD	NM_000285.4	23287645
PIK3CA	NM_006218.4	PMID: 20197055
PIK3R1	NM_181523.3	PMID: 29893513
PLAGL1	NM_001080951.3	PMID: 10655556
PLIN1	NM_002666.5	PMID: 21345103
POMC	NM_001035256.3	PMID: 17728716
PON1	NM_000446.7	PMID: 15853770/ PMID: 25964115
PPARG	NM_015869.5	PMID: 25584717
PPP1R3A	NM_002711.4	PMID: 12118251
PTEN	NM_000314.8	PMID: 22970944
PTF1A	NM_178161.3	PMID: 32893856/PMID: 15543146
PTPRD	NM_002839.4	25765181
RFX6	NM_173560.4	34416793/PMID: 29026101
SGCG	NM_000231.3	PMID: 28123479/31931849
SH2B1	NM_015503.2	PMID: 32547144
SIRT1	NM_012238.5	PMID: 26637398/PMID: 33577446
SIX3	NM_005413.4	29621232

SLC16A1	NM_001166496.2	PMID: 28666119
SLC19A2	NM_006996.3	PMID: 28004468
SLC29A3	NM_001174098.2	PMID: 23623699
SLC2A2	NM_000340.2	PMID: 27500523
SLC30A8	NM_001172813.2	PMID: 30319545
SOD2	NM_000636.4	PMID: 31524543
SOX2	NM_003106.4	PMID: 33294436
SOX9	NM_000346.4	PMID: 26733677
SPINK1	NM_003122.5	PMID: 32094658
SPRY2	NM_005842.4	PMID: 21909839
SRR	NM_021947.3	PMID: 24843776/ PMID: 28869590
SREBF1	NM_001005291.3	PMID: 33577446
STAT3	NM_139276.3	PMID: 25038750
TBC1D4	NM_014832.5	PMID: 25043022
TCF7L2	NM_001146274.2	PMID: 29100364
THADA	NM_001083953.2	19833888
TRIM32	NM_012210.4	PMID: 16606853/PMID: 24041679/PMID: 17994549
TTC8	NM_001288781.1	PMID: 20451172/PMID: 19402160
UCP2	NM_003355.3	PMID: 11440717
VEGFA	NM_001025366.3	PMID: 29533820
WFS1	NM_006005.3	PMID: 33693650/PMID: 34006618
WRN	NM_000553.6	PMID: 32320127
ZFP57	NM_001109809.5	PMID: 18622393/
APPL1	NM_012096.3	PMID: 26073777
BBS1	NM_024649.5	PMID: 12118255
BBS2	NM_031885.5	PMID: 11285252
BBS4	NM_033028.5	26518167
BBS5	NM_152384.3	30850397
BBS7	NM_176824.3	PMID: 19093007
BBS10	NM_024685.4	PMID: 25439097/28808579
BBS12	NM_152618.3	24849935
COQ2	NM_001358921.2	PMID: 19375058
COQ9	NM_020312.4	26081641
CTLA4	NM_005214.5	PMID: 31156616/ PMID: 25329329
DNAJC3	NM_006260.5	PMID: 25466870
DUT	NM_001025248.2	PMID: 28073829

DYRK1B	NM_004714.3	PMID: 24827035
EIF2S3	NM_001415.4	PMID: 32180185/PMID: 30878599
HNF1A	NM_000545.8	34202200/PMID: 34035238
HNF1B	NM_000458.4	32756155
HNF4A	NM_000457.6	PMID: 15735891/32670997
ITCH	NM_031483.7	PMID: 20170897
NEXMIF	NM_001008537.3	PMID: 27358180
LPL	NM_000545.8	PMID: 16896939
LRBA	NM_001364905.1	PMID: 28601686
MAFA	NM_201589.4	PMID: 29339498
MDH2	NM_005918.4	27989324
MKKS/BBS6	NM_170784.3	PMID: 10973251/PMID: 19247371
MKS1/BBS13	NM_017777.4	24608809/27570071
MYO5A	NM_001382347.1	PMID: 29670293
NKX2-2	NM_002509.4	PMID: 24411943
NKX6-1	NM_006168.3	PubMed:21544516/PubMed:29439679
PCBD1	NM_000281.4	PMID: 24204001/PMID: 33749890
LZTFL1	NM_001276378	PubMed: 17163542
POLD1	NM_002691.4	34517090/PMID: 26350127
PPP1R15B	NM_032833.5	PMID: 26307080
SDCCAG8/BBS16	NM_006642.5	32926352
CAVIN1	NM_012232.6	27717241
CDKN1C	NM_012232.6	PMID: 15888726/
FOS	NM_005252.4	
GCK	NM_000162.5	PMID: 9662401/ 33812904

These genes are involved in glucose metabolism, known to cause monogenic diabetes or associated syndromes, and found from genome-wide association data of type 2 diabetes. The molecular function and the Human Phenotype Ontology of each gene has been confirmed according to the VarSome Database (https://varsome.com/).

Table S2. Sanger sequencing results among the 11 Tunisian family members

Patient ID	Available family members	Variant Nomenclature	Genotype	Sanger sequencing result
F1	P1	*ABCC8:* c.250G>Ap.Val84Ile	Het	Presence of the variant
		KANK1: c.793G>A p. Glu265Lys	Hom	Presence of the variant
F2	P2	*PPP1R3A*: c.2267C>Tp.P756L	Het	Presence of the variant
		RFX6: c.1733G>C p.R578P	Het	Presence of the variant
	Diabetic mother	*PPP1R3A*: c.2267C>Tp.P756L	Hom	Absence of the variant
		RFX6: c.1733G>C p.R578P	Het	Presence of the variant
	Non-diabetic brother	*PPP1R3A*: c.2267C>Tp.P756L	Hom	Absence of the variant
		RFX6: c.1733G>C p.R578P	Hom	Absence of the variant
F3	P3	*UCP2*: c.382G>A:p.A128T	Het	Presence of the variant
		FN3K: c.101A>G:p.D34G	Het	Presence of the variant
	Non-diabetic father	*UCP2*: c.382G>A:p.A128T	Het	Presence of the variant
		FN3K: c.101A>G:p.D34G	Het	Presence of the variant
F4	P4	*BBS12*:c.355G>A:p.G119S	Hom	Presence of the variant
		TTC8:c.194A>G:p.D65G	Het	Presence of the variant
	Non-diabetic mother	*BBS12*:c.355G>A:p.G119S	Het	Presence of the variant
		TTC8:c.194A>G:p.D65G	Hom	Absence of the variant
	Non-diabetic sister	*BBS12*:c.355G>A:p.G119S	Hom	Presence of the variant
		TTC8:c.194A>G:p.D65G	Hom	Absence of the mutation
F5	P5	*KANK1*: c.1652G>A : p. C551Y	Het	Presence of the variant
	Diabetic father	*KANK1*: c.1652G>A : p. C551Y	Het	Presence of the variant
	Non-diabetic mother	KANK1: c.1652G>A : p. C551Y	Hom	Absence of the variant
F6	P6	*TTC8*:c.194A>G:p.D65G	Het	Presence of the variant
	Non-diabetic father	*TTC8*:c.194A>G:p.D65G	Het	Presence of the variant
	Non-diabetic mother	*TTC8:*c.194A>G:p.D65G	Hom	Absence of the variant

F7	P7	*GCKR*:c.316G>A:p.G106R	Het	Presence of the variant
		PPP1R3A:c.2267C>T:p.P756L	Hom	Presence of the variant
	Non-diabetic father	*GCKR*:c.316G>A:p.G106R	Het	Presence of the variant
		PPP1R3A:c.2267C>T:p.P756L	Het	Presence of the variant
	Non-diabetic mother	*GCKR*:c.316G>A:p.G106R	Hom	Absence of the variant
		PPP1R3A:c.2267C>T:p.P756L	Het	Presence of the variant
F8	P8	*ALMS1*:c.9617C>T:p.T3206I	Het	Presence of the variant
		ALMS1: c.1448G>C : p.Gly483Ala	Het	Presence of the variant
	Non-diabetic mother	*ALMS1*:c.9617C>T:p.T3206I	Het	Presence of the variant
		ALMS1: c.1448G>C : p.Gly483Ala	Hom	Absence of the variant
F9	P9	*KLF11*:c.1382G>A:p.R461Q	Het	Presence of the variant
		WFS1:c.2206G>A:p.G736S	Het	Presence of the variant
	Non-diabetic father	*KLF11*:c.1382G>A:p.R461Q	Hom	Absence of the variant
		WFS1:c.2206G>A:p.G736S	Hom	Absence of the variant
	Diabetic mother	*KLF1*:c.1382G>A:p.R461Q	Het	Presence of the variant
		WFS1:c.2206G>A:p.G736S	Het	Presence of the variant
F10	P10	*INSR*:c.1649C>T:p.A550V	Het	Presence of the variant
	Diabetic father	*INSR*:c.1649C>T:p.A550V	Het	Presence of the variant
F11	P11	*ALMS1*: c.41_42ins GGA:p.E14delinsEE	Het	Presence of the variant
		PDX1: c.226G>A: p.D76N	Het	Presence of the variant

Het: Heterozygous state, Hom: Homozygous state.

2. Supplementary Figures

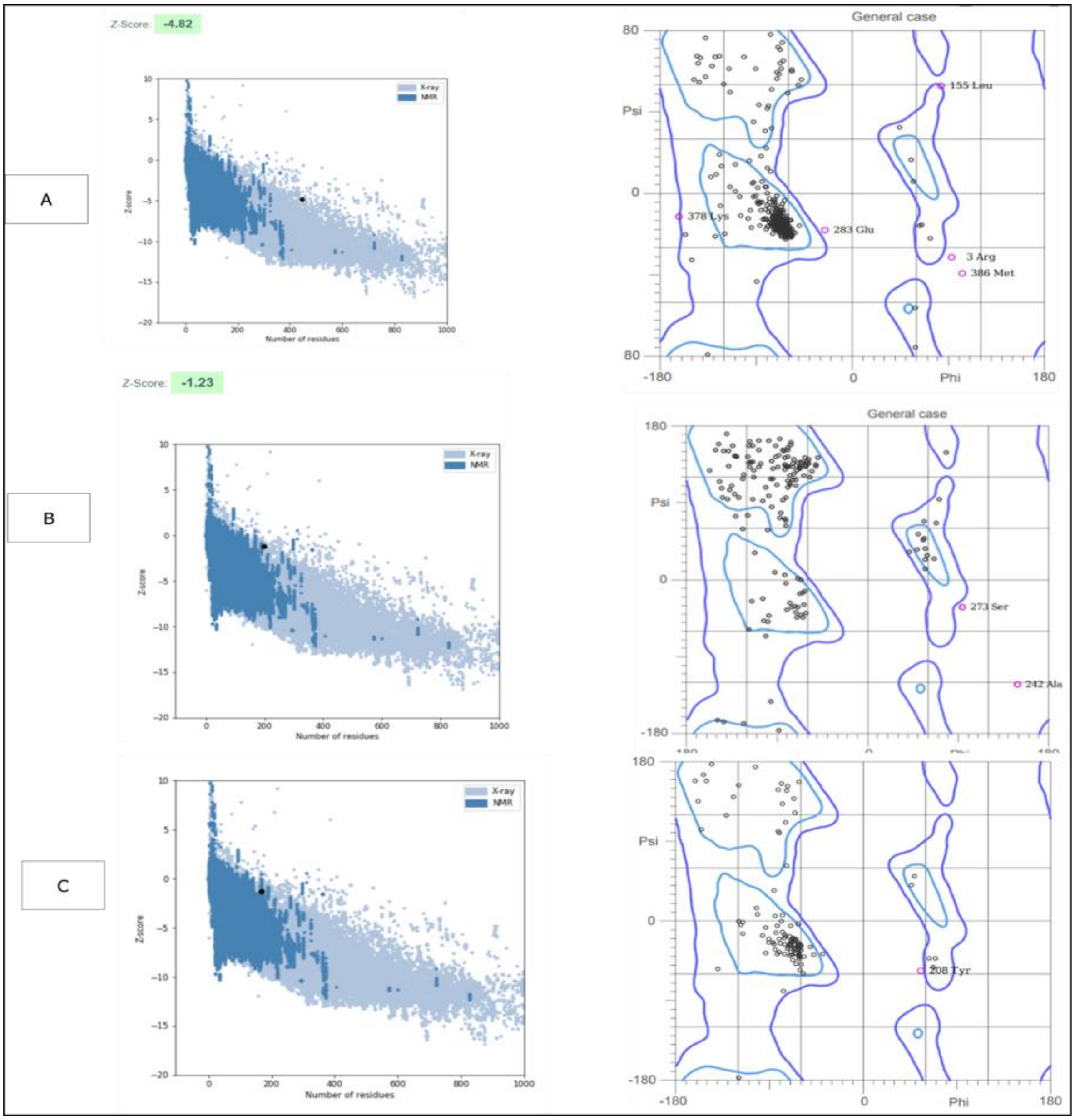

Figure S1: Predicted models quality assessment: Z-score and Ramachandran plot distribution for the predicted models.

A: KANK1 protein (aa:180-626). B: ALMS1 protein (aa: 322-521). C: ALMS1 protein (aa: 3162-3331).

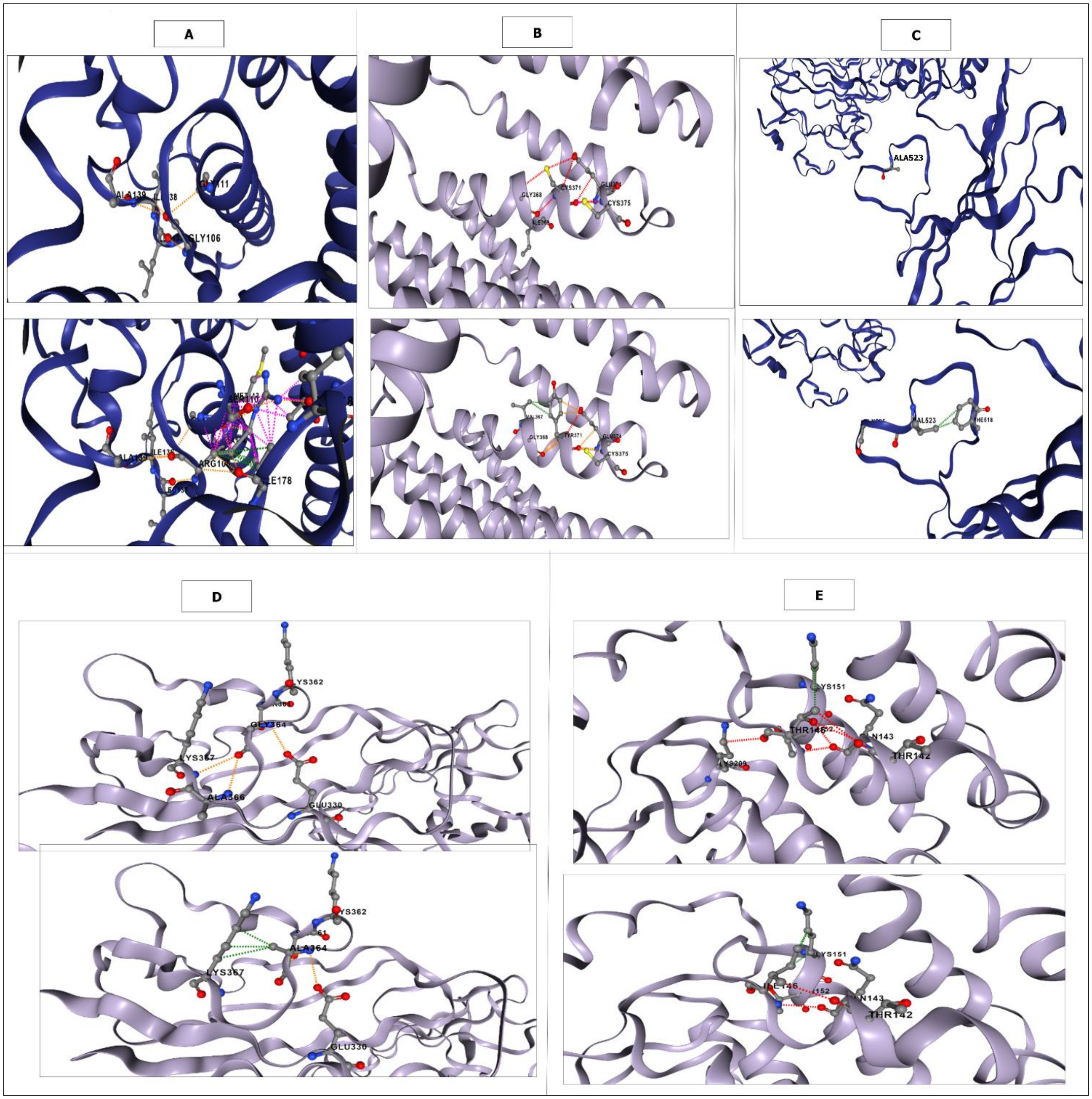

Figure S2: Structural analyses results of some identified genetic variants.

A: Structural analysis of the GCKR p.Gly106Arg variant. B: Structural analysis of the KANK1 p. Cys551Tyr variant, the amino acid Cys551 corresponds to Cys371 in our model. C: Structural analysis of the INSR p.Ala550Val variant, the amino acid Ala550 corresponds to Ala523 in the crystalized INSR protein. D: Structural analysis of the ALMS1 p.Gly483Ala variant, Gly483 corresponds to the amino acid Gly364 in our ALMS1 model. E: Structural analysis of the ALMS1 p.Thr3206Ile variant. Thr3206 corresponds to the amino acid Thr146 in the ALMS1 predicted model. Red dashes represent hydrogen bonds, orange dashes represent polar interactions, green dashes represent polar interaction, and pink dashes represent steric clash.
